# Supplementary material for: Applying dyadic digital psychological interventions for reducing caregiver burden in the illness context: a systematic review and a meta-analysis protocol
Source: BMJ Open. 2023 May 10;13(5):e070279. doi: 10.1136/bmjopen-2022-070279 (PMC10173984; doi:10.1136/bmjopen-2022-070279)
Supplement: Supplementary data [file bmjopen-2022-070279supp002.pdf]

Applying dyadic digital psychological interventions for reducing caregiver burden in the illness context: A systematic review and a meta-analysis protocol

The full search strategy that will be further adapted to the following databases: PubMed, Embase, the Cochrane Library, Cinhal, Scopus, PsycINFO, and MEDLINE.

**Concept Category (combined with AND)**	**Search terms (combined with OR)**
Dyads	dyad, dyads, dyadic, couple, couples, spouse, spouses, "Spouse" [MeSH], "Family" [MeSH], informal caregiver, caregiver, "Caregivers" [MeSH], carer, carers, partner
Digital	Digital, Online, Internet, internet-based, "Internet"[MeSH], cyberspace, web, web-based, ehealth, e-health, "Telemedicine"[MeSH], mobile health, mhealth, m-health, social media, "Social Media"[MeSH], blog, blogs, mobile app, mobile application, User-Computer Interface, website, webpage
Psychological intervention	psychosocial intervention, behavior therapy, "Behavior Therapy" [MeSH], cognitive therapy, "Cognitive Behavioral Therapy" [MeSH], couples therapy, "Couples Therapy" [MeSH], family therapy, "Family Therapy" [MeSH], psychoeducation, psycho-education, psychoeducational, psycho-educational, "Psychology, Medical" [MeSH], "Psychology, Clinical" [MeSH]

(Dyad* OR Couple* OR "Informal caregiver*" OR Spouse* OR Partner* OR Carer* OR "Care partner*" OR "Informal Carer*") **AND** (Digital OR Online OR "Interne-based" OR Internet OR "Web-based" OR ehealth OR mhealth OR "e-health" OR "m-health" OR "mobile app" OR website OR webpage OR "User-computer Interface") **AND** (Intervention* OR Program* OR Therap* OR Psych* OR "Self-help" OR Support* OR "family-therapy" OR "Cognitive Behavioral Therapy" OR "Behaviour Therapy" or Psychoeducational OR "Psycho-education" OR "Clinical psychology" OR Rehabilitation* OR "Couples therap*") **AND** (RCT OR "randomized controlled trial*) **AND** ("caregiver burden") **NOT** (Parent* OR Child* OR Nurse* OR "Formal caregiv*" OR "Formal carer*")
