# Supplementary material for: Applying dyadic digital psychological interventions for reducing caregiver burden in the illness context: a systematic review and a meta-analysis protocol
Source: BMJ Open. 2023 May 10;13(5):e070279. doi: 10.1136/bmjopen-2022-070279 (PMC10173984; doi:10.1136/bmjopen-2022-070279)
Supplement: Author's manuscript [file bmjopen-2022-070279.draft_revisions.pdf]

BMJ Open

BMJ Open is committed to open peer review. As part of this commitment we make the peer review history of every article we publish publicly available.

When an article is published we post the peer reviewers' comments and the authors' responses online. We also post the versions of the paper that were used during peer review. These are the versions that the peer review comments apply to.

The versions of the paper that follow are the versions that were submitted during the peer review process. They are not the versions of record or the final published versions. They should not be cited or distributed as the published version of this manuscript.

BMJ Open is an open access journal and the full, final, typeset and author-corrected version of record of the manuscript is available on our site with no access controls, subscription charges or pay-per-view fees (http://bmjopen.bmj.com).

If you have any questions on BMJ Open's open peer review process please email info.bmjopen@bmj.com

BMJ Open

# Applying dyadic digital psychological interventions for reducing caregiver burden in the illness context: A systematic review and a meta-analysis protocol

| | |
|---|---|
| Journal: | *BMJ Open* |
| Manuscript ID | bmjopen-2022-070279 |
| Article Type: | Protocol |
| Date Submitted by the Author: | 21-Nov-2022 |
| Complete List of Authors: | Semonella, Michelle; Bar-Ilan University, Department of Psychology, Bar-Ilan University 5290002, Israel<br>Bertuzzi, Vanessa; Università Cattolica del Sacro Cuore, Psicologia<br>Dekel, Rachel; Bar-Ilan University, School of Social Work<br>Andersson, Gerhard; Linkoping University,<br>Pietrabissa, Giada; IRCCS Istituto Auxologico Italiano, Ospedale San Giuseppe, Psychology Research Laboratory; Universita Cattolica del Sacro Cuore, Department of Psychology<br>Vilchinsky, Noa; Universitat Bar-Ilan, Department of Psychology |
| Keywords: | MENTAL HEALTH, Quality in health care < HEALTH SERVICES ADMINISTRATION & MANAGEMENT, Protocols & guidelines < HEALTH SERVICES ADMINISTRATION & MANAGEMENT |
| | |

SCHOLARONE™
Manuscripts

# Applying dyadic digital psychological interventions for reducing caregiver burden in the illness context: A systematic review and a meta-analysis protocol

Michelle Semonella[1]; Vanessa Bertuzzi[2]; Rachel Dekel[3]; Gerhard Andersson[4,5,6]; Giada Pietrabissa[2,7]; Noa Vilchinsky[1]

[1]Department of Psychology, Bar-Ilan University, Ramat Gan, Israel
[2]Department of Psychology, Catholic University of Milan, Milan, Italy
[3]School of Social Work, Bar-Ilan University, Ramat Gan, Israel
[4]Department of Behavioral Sciences and Learning, Linköping University, Linköping, Sweden
[5]Department of Biomedical and Clinical Sciences, Linköping University, Linköping, Sweden
[6]Department of Clinical Neuroscience, Karolinska Institute, Stockholm, Sweden
[7] Psychology Research Laboratory, IRCCS Istituto Auxologico Italiano, Milan, Italy

Corresponding Author: Michelle Semonella; michelle.semonella@biu.ac.il

Keywords: informal caregivers; digital psychological interventions; caregiver burden; dyadic interventions; eHealth; systematic review; meta-analysis



For peer review only - http://bmjopen.bmj.com/site/about/guidelines.xhtml

*Abstract*

**Introduction:** Providing informal care to one's romantic partner who is ill may become a highly distressing and demanding task. Based on the innovative dyadic coping model, several support interventions have been developed to alleviate informal caregivers' burden, taking into account both caregivers' and care receivers' needs. Considering the unique challenges characterizing the caregiving phenomenon, such as geographical barriers and time restrictions, digital solutions should be considered. However, there is a lack of research examining the effectiveness of dyadic digital solutions. Thus, this review aims to examine the existing literature on the efficacy of dyadic digital psychological interventions designed for caregivers and their care-receivers couples within the illness context.

**Methods and analysis:** Randomized controlled trials targeting caregivers' burden among dyads of informal caregivers and care-receivers will be identified via an electronic search of the following databases: PubMed, Scopus, PsycINFO, MEDLINE, and supplemented by hand searching of previous systematic reviews. The search will be undertaken following the PICO elements. If possible, a meta-analysis will be conducted to examine: 1. The effectiveness of dyadic digital psychological interventions for reducing caregivers' burden (primary outcome) among informal caregivers who are in a romantic relationship with the care receivers; 2. The effectiveness of dyadic digital psychological interventions on secondary outcomes such as anxiety, depression, stress, quality of life, well-being, and self-efficacy among informal caregivers and care receivers; 3. Moderating effects of clinical and methodological factors on caregivers' burden. Prior to inclusion in the review, retrieved papers will be critically appraised by two independent reviewers. The Cochrane Risk of Bias tool will assess the risk of bias for randomized controlled trials.



For peer review only - http://bmjopen.bmj.com/site/about/guidelines.xhtml

**Ethics and dissemination:** Ethical approval is not required as no primary data will be collected. Findings will be disseminated through peer-reviewed publications, presentations at academic conferences, and lay summaries for various stakeholders.

**PROSPERO registration number** CRD42022299125

For peer review only



For peer review only - http://bmjopen.bmj.com/site/about/guidelines.xhtml

Strengths and limitations of this study

- **To our best knowledge, this is the first comprehensive systematic review of dyadic digital psychological interventions for reducing caregiver burden, tapping diverse illness contexts, and synthesizing data regarding diverse digital tools.**
- **The review will guide the development of tailored digital dyadic interventions to support caregivers.**
- **Only randomized controlled trials will be included to maintain a high methodological standard.**
- **The studies may not have collected all the variables of interest. Thus, we might not be able to examine all candidate moderators.**
- **The review will be restricted to studies published in English, which might be indicative of a language bias.**

For peer review only



For peer review only - http://bmjopen.bmj.com/site/about/guidelines.xhtml

## INTRODUCTION

### *Informal caregiving*

Informal care is defined as the unpaid care provided to an older, frail, or ill person, by a person such as a spouse, parent, child, another relative, neighbor, friend, or other non-kin [1]. The phenomenon of providing informal care is growing, mostly due to the growth in the aging population, the increasing prevalence of chronic illnesses and more effective medical treatments saving lives. To date, it is estimated that 44 million people in Europe provide informal care to family or friends [2]. Indeed, In Europe, 80% of Long-Term Care (LCT) for non-independent people is provided by informal caregivers; this estimation makes them an essential element of the care system in Europe [3]. At the same time, the growing number of caregivers has made caregiving a public health issue [4, 5]. Being an informal caregiver can be a stressful experience and is often associated with negative psychosocial and health consequences as well as financial strains, all of which could increase caregiving burden [6, 7]. Caregiver burden is a multidimensional concept consisting of physical, psychological, emotional, social, and financial stressors linked with the caregiving experience which might contribute to caregivers' physical and psychological illnesses [8-10]. Indeed, due to their extensive caregiving responsibilities, many caregivers report physical exhaustion [11] and even negative health consequences such as increased cardiovascular reactivity [12] and poor

For peer review only

immune system response [13-15]. It is therefore necessary to find ways to alleviate caregivers' burden and distress by developing suitable and feasible psychological support interventions or implementing existing ones. When designing or implementing interventions it is important to take into account the characteristics, limitations, and features that characterizes informal care, such as its dyadic nature and caregivers' difficulties in terms of lack of time and money.

***Novel approaches to interventions for caregivers: the call for dyadic and digital solutions***

During the last twenty years, there has been a shift in perspective in the health psychology literature: from an individual perspective of coping with the illness to a dyadic one [16-18]. Researchers have demonstrated that both members of the dyad actively share resources and manage stressors together as a couple. Specifically, a new conceptualization has emerged viewing illness as a “we-disease” [19], which taps mutual influences between two coping partners in a dyad [20-22]. Particularly, providing care in romantic relationships differs from other kinds of relationships for three main reasons. First, romantic partners are most likely to live together with the care recipients, and therefore usually provide support when needed. Second, caregivers who are in a romantic relationship are usually older than adult children who provide care for their parents, thus they may have their own health problems which may lead to greater perceived stress while providing informal care. Third, the romantic partner is usually the most important attachment figure for the care receiver [23], and many expectations



For peer review only - http://bmjopen.bmj.com/site/about/guidelines.xhtml

are directed toward him/her on behalf of the patient [24, 25]. Thus, caregiving spouses in particular might be at higher risk for caregiver burden [26]. Thus, given that caregiving is conceptualized as a dyadic process rather than an individualized one [16], any intervention developed for caregivers should be designed dyadically, taking into account both caregivers' and care receivers' needs and consequences, as well as the relationship between the two. Indeed, different dyadic interventions have been developed to ease caregivers' stress and help them better cope with their condition in various health contexts; and they are effective in improving outcomes (such as caregiver burden, quality of life, stress, anxiety, depression) in both informal caregivers and care-receivers [27-30].

Although dyadic interventions were shown to be successful and beneficial for both members of the dyad [31-34], most of them have been delivered in person, requiring the participation of both members which may limit their ultimate scalability and accessibility [35]. It is, therefore, necessary to develop interventions that are dyadic in nature – involving both partners – while at the same time being feasible. In recent years using information technology has become a regular part of our daily life, and also common in the clinical field and health care. During the coronavirus pandemic, psychological support delivered through digital solutions became more relevant, accepted, and feasible [36]. Indeed, the Covid-19 outbreak facilitated the acceptability and willingness to use information technology tools to deliver psychological



For peer review only - http://bmjopen.bmj.com/site/about/guidelines.xhtml

interventions [37]. Thanks to psychologically-based digital interventions, it is possible to reach patients from a distance and research suggests that this mode of delivery can be as effective as other treatment formats [38], and serve as a complement or even alternative to regular face-to-face treatments [39]. Digital interventions would be particularly useful and efficient to fill gaps due to caregiving conditions, as they suit caregivers' needs in terms of lack of time, money, and distance [37]. Moreover, internet interventions can have beneficial effects on caregivers' mental health, including reduction of depression, stress, anxiety, and caregiver burden [40, 41], and have also been found to increase self-efficacy, self-esteem, and strain of caregivers of adults with chronic conditions [42] as well as enhance the sense of competence, coping skills and strategies, and quality of life in caregivers of older adults [43]. Thus, interventions that integrate the dyadic approach with technology could be found most suitable to meet caregivers' needs, as well as feasible and cost-effective. As the digital-dyadic approach became more prevalent, it is first important to review the state-of-art of the efficacy of dyadic digital psychological interventions for informal caregivers and care-receivers in a romantic relationship.

**Aim of the study**

To the best of our knowledge, no review or meta-analysis has systematically evaluated the current research on dyadic digital psychological interventions for informal caregivers. This



For peer review only - http://bmjopen.bmj.com/site/about/guidelines.xhtml

study aims to systematically review the existing literature on the efficacy of dyadic digital psychological interventions designed for couples of informal caregivers and their care-receivers within the illness context.

Specifically, we aim to examine:

1. The efficacy of dyadic digital psychological interventions for reducing caregivers' burden among informal caregivers who are in a romantic relationship with adults with a chronic illness;
2. The effectiveness of dyadic digital psychological interventions on secondary outcomes such as anxiety, depression, stress, quality of life, well-being, self-efficacy, and relationship satisfaction among informal caregivers and care-receivers;
3. The moderating effects of clinical and methodological factors on caregivers' burden.

## METHODS AND ANALYSIS

This protocol is guided by the Preferred Items for Reporting of Systematic Reviews and Meta-Analyses Protocol (PRISMA) checklist [44] (online supplement appendix 1) and the Cochrane Risk of Bias tool to assess the risk of bias for randomized controlled trials [45].

### Study registration

Following the PRISMA guidelines [44], our systematic review protocol was registered with the International Prospective Register of Systematic Reviews (PROSPERO) on March 3rd, 2022



For peer review only - http://bmjopen.bmj.com/site/about/guidelines.xhtml

(registration number CRD42022299125). Any important protocol amendments will be recorded in PROSPERO and published with the results of the review.

**Eligibility criteria**

Inclusion and exclusion criteria are categorized by population, interventions, comparators, outcomes, and study design (PICOS) [46].

Eligible studies for inclusion will be in English language peer-reviewed studies or unpublished studies such as doctoral thesis to reduce publication bias.

**Population**

We will include studies examining adults (18 years or older) who provide informal care and their care-receivers in a romantic relationship. Thus, we will not include studies that include formal caregivers; studies in which the sample included caregiving parents, siblings, or other non-romantic relationships; and finally, studies that do not collect data on both informal caregivers and care-receivers. No restrictions have been placed on participants' gender and ethnicity.

**Interventions**

Any psychological, psychoeducational, psychosocial, or combination of psychological interventions using specific therapeutic principles and techniques, delivered via a technological tool (e.g., computer, telephone, videos). Any other type of interventions such as pharmacological interventions, acupuncture, smoking cessation, etc. will be excluded. All the interventions included must be dyadic interventions addressing, targeting, and providing data



For peer review only - http://bmjopen.bmj.com/site/about/guidelines.xhtml

on both informal caregivers and care-receivers. Individual intervention or studies that provide data on either caregiver or care-receiver only will not be included in the current review.

## Comparator

Both active and inactive comparators will be eligible, such as no intervention, usual care, waiting list,

and attention control.

## Outcomes

Primary outcomes

Studies will be included only if one or more standardized measurements of caregivers' burden have been used.

Secondary outcomes

Secondary outcomes of interest are quantitative measurements listed below:

- Anxiety;
- Depression;
- Stress;
- Quality of life;
- Well-being;
- Self-efficacy.

## Study design

We will include only randomized controlled trials, to optimize the internal and external validity of this review.

For peer review only

## Information sources

## Search strategy

From November 1st to December 1st, 2021, two reviewers (MS and VB) independently searched the following database for relevant articles: PubMed, Scopus, PsycINFO, and MEDLINE.

For each database, a comprehensive search strategy was developed under the PICO (Population, Intervention, Comparison, and Outcome) framework [46]. Boolean and truncation operators were used to systematically combine search terms and to screen for a list of pre-specified search terms in titles and abstracts.

No restrictions were placed on the publication period. The reference lists of all selected articles and relevant systematic review will be manually screened to identify further relevant records for possible inclusion [47, 48]

Additionally, studies from grey literature (e.g., thesis dissertation, conference papers/abstract) will be searched in OpenGrey (http://www. Open grey. eu/) and will be included in case they fulfill the inclusion criteria.

Data will be exported to Endnote X8 (Clarivate Analytics, Philadelphia, Pennsylvania, USA) and Excel for the title and abstract screening.

## Study records

Data management

Data will be imported into Endnote X8 (Clarivate Analytics, Philadelphia, Pennsylvania, USA), while (if possible), Stata V.16.0 (StataCorp) will be used for meta-analysis.



For peer review only - http://bmjopen.bmj.com/site/about/guidelines.xhtml

Selection process

Two reviewers will independently screen study titles and abstracts retrieved from searches; they will perform full paper checks of identified potentially eligible studies. The PICOS criteria will be used to select eligible studies. In the results manuscript will be documented and reported the overall reasons for exclusion at the full-text screening, using a PRISMA flow chart. A detailed overview of the reasons for the inclusion/exclusion of studies at the PICOS item level will be presented in a table in the results manuscript. Disagreements between reviewers will be solved by consulting a third reviewer. The overall selection process will be represented as a PRIMA flow diagram.

**Data extraction**

All reviewers will be involved in reaching a consensus on the data to be extracted and the terminology used before the start of the screening. The selection process of the relevant studies will be conducted by two independent reviewers (MS and VB). One reviewer (MS) will screen the titles of each study based on the eligibility criteria and when these are deemed relevant, she will go through abstracts. Once narrowed down by abstracts, a full-text review process will be completed in duplicate by two reviewers (MS and VB) for studies that met the eligibility criteria at screening and for studies with unclear relevance. If there are any disagreements, a third independent reviewer (either NV or RD) will be consulted to resolve discrepancies. Original authors of studies identified will be contacted if the full-text paper was not available or the relevance of a paper was unclear.



For peer review only - http://bmjopen.bmj.com/site/about/guidelines.xhtml

### Risk of bias

The Cochrane Collaboration's Risk for Bias tool will be used to evaluate the risk of bias and the quality of the studies in the form of randomized controlled trial [45]. Thanks to this tool each domain of potential bias will be classified as "low risk" or "unclear risk" or "high risk" [49].

Two researchers will work independently. Any disagreement will be solved by a third reviewer.

### Data synthesis

A narrative synthesis will be undertaken to present and explain the findings. Two reviewers (MS, VB) will independently read the selected studies and will propose an initial framework for its synthesis. These frameworks will serve as a basis for a consensus synthesis framework, decided by all reviewers in a group discussion. All reviewers will be involved in writing the narrative synthesis, following a joint iterative process of organizing and cross-validating the reported results.

Meta-analysis

If data allow, a meta-analysis will be performed to explore the effectiveness of dyadic digital psychological interventions for reducing caregivers' burden among informal caregivers by calculating post-treatment between-group standardized mean effect sizes for the primary outcome using Hedges' g. In addition, the effectiveness of dyadic digital psychological interventions will be examined on selected secondary outcomes (e.g., depression, anxiety, stress, quality of life, well-being, and self-efficacy) among both informal caregivers and/or care-receivers.

Dichotomous outcomes will be measured by risk ratio (RR) and its 95% confidence interval. Continuous outcomes will be measured by calculating the mean difference (MD) with 95% CI



For peer review only - http://bmjopen.bmj.com/site/about/guidelines.xhtml

when the studies use the same instrument for assessing the outcome. The standardized mean difference will be used when studies use different instruments.

Assessment of heterogeneity

A random-effect model will be performed for all the analyses because of the potential heterogeneity among clinical trial results. The heterogeneity between-study will be measured using the Cochrane's test of heterogeneity (Q) and will be reported using $I^2$ statistics, alongside CIs [50, 51]. The indirect comparisons between technology will be assessed using the Bucher and Glenny method [52].

Moderator analyses

Prespecified subgroup analyses will be performed to assess the potential association of some clinical factors with the primary outcome. Subgroups were defined according to toa. Study quality characteristics: control type (active VS non-active); b. Informal caregivers characteristics: age, women percentage, and education; c. Patient characteristics: age, women percentage, education, type of illness; d. Intervention characteristics: number of sessions, duration, type of therapy (e.g., CBT VS mindfulness), modality (tool used to deliver the intervention (e.g., telephone VS video) [53].

Dealing with missing data

Intention-to-treat data will be used when possible [54].

**Confidence in cumulative evidence**

The Grading of Recommendations Assessment, Development, and Evaluation (GRADE) [55, 56] tool will be used to assess the certainty of the evidence of both primary outcome and



For peer review only - http://bmjopen.bmj.com/site/about/guidelines.xhtml

secondary outcomes as “high”, “moderate”, “low” or “very low”, according to the GRADE approach. A summary of evidence will be presented in a table.

## Ethics and dissemination

Any identifiable patient data will be excluded; thus, ethical approval and participant consent are not required. Findings will be disseminated through peer-reviewed publications, presentations at academic conferences, and lay summaries for various stakeholders.

## Contributors

MS contributed to the conception and design of the study and wrote the manuscript. NV contributed to the conception and design of the study and critically revised the manuscript drafts. VB, GA, RD, and GP critically revised the study design, assisted in data synthesis and analysis, and revised the manuscript’s final draft. All authors approved the final manuscript.

## Funding

This work is funded by the European Union’s Horizon 2020 research and innovation program under the Marie-Sklodowska Curie grant agreement no 814072.

## Competing interests

None declared.



For peer review only - http://bmjopen.bmj.com/site/about/guidelines.xhtml

## References

1. Triantafillou, J., et al., *Informal care in the long-term care system European overview paper.* 2010.
2. de Lanchy, G., N. Lorentz, and K. Leduc, *Long term care workforce: Employment and working conditions-Luxembourg*, in *Long term care workforce: Employment and working conditions*. 2020, Publications Office of the European Commission.
3. Naiditch, M., et al., *User perspectives in long-term care and the role of informal carers*, in *Long-Term Care in Europe*. 2013, Springer. p. 45-80.
4. Schulz, R. and T.L. Patterson, *Caregiving in geriatric psychiatry.* The American Journal of Geriatric Psychiatry, 2004. **12**(3): p. 234-237.
5. Edwards, V.J., et al., *Characteristics and Health Status of Informal Unpaid Caregivers - 44 States, District of Columbia, and Puerto Rico, 2015-2017.* MMWR. Morbidity and mortality weekly report, 2020. **69**(7): p. 183-188.
6. Hwang, B., et al., *Caregiving for patients with heart failure: impact on patients' families.* Am J Crit Care, 2011. **20**(6): p. 431-41; quiz 442.
7. Metzelthin, S.F., et al., *Positive and negative outcomes of informal caregiving at home and in institutionalised long-term care: a cross-sectional study.* BMC geriatrics, 2017. **17**(1): p. 232.
8. Jones, C., *Careers in project networks: The case of the film industry.* The boundaryless career: A new employment principle for a new organizational era, 1996. **58**: p. 58-75.
9. Li, Q.P., Y.W. Mak, and A.Y. Loke, *Spouses' experience of caregiving for cancer patients: a literature review.* Int Nurs Rev, 2013. **60**(2): p. 178-87.
10. Pottie, C.G., et al., *Informal caregiving of hospice patients.* J Palliat Med, 2014. **17**(7): p. 845-56.
11. Pressler, S.J., et al., *Family caregiver outcomes in heart failure.* Am J Crit Care, 2009. **18**(2): p. 149-59.
12. Vitaliano, P.P., J. Zhang, and J.M. Scanlan, *Is caregiving hazardous to one's physical health? A meta-analysis.* Psychological bulletin, 2003. **129**(6): p. 946.
13. Kiecolt-Glaser, J.K., et al., *Spousal caregivers of dementia victims: longitudinal changes in immunity and health.* Psychosomatic medicine, 1991. **53**(4): p. 345-362.
14. Laudenslager, M.L., *"Anatomy of an Illness": control from a caregiver's perspective.* Brain Behav Immun, 2014. **36**: p. 1-8.
15. Lutgendorf, S.K. and M.L. Laudenslager, *Care of the caregiver: stress and dysregulation of inflammatory control in cancer caregivers.* 2009, American Society of Clinical Oncology.
16. Revenson, T.A., et al., *Caregiving as a dyadic process*, in *Caregiving in the illness context*. 2016, Springer. p. 25-37.
17. Gouin, J.-P., et al., *Dyadic coping and inflammation in the context of chronic stress.* Health Psychology, 2016. **35**(10): p. 1081.
18. Carr, R., et al., *Dyadic interventions to promote physical activity and reduce sedentary behaviour: Systematic review and meta-analysis.* Health Psychology Review, 2019. **13**(1): p. 91-109.
19. Kayser, K., L.E. Watson, and J.T. Andrade, *Cancer as a" we-disease": Examining the process of coping from a relational perspective.* Families, Systems, & Health, 2007. **25**(4): p. 404.
20. Acitelli, L.K. and H.J. Badr, *My Illness or Our Illness? Attending to the Relationship When One Partner Is Ill*, in *Couples coping with stress: Emerging perspectives on dyadic coping.* 2005, American Psychological Association: Washington, DC, US. p. 121-136.



For peer review only - http://bmjopen.bmj.com/site/about/guidelines.xhtml

21. Rohrbaugh, M.J., et al., *Prognostic significance of spouse we talk in couples coping with heart failure.* Journal of Consulting and Clinical Psychology, 2008. **76**(5): p. 781-789.
22. Revenson, T.A., et al., *Interventions to support caregivers*, in *Caregiving in the illness context*. 2016, Springer. p. 90-104.
23. Pinquart, M. and S. Sörensen, *Spouses, adult children, and children-in-law as caregivers of older adults: a meta-analytic comparison.* Psychology and aging, 2011. **26**(1): p. 1.
24. Cash, B., J. Warburton, and S. Hodgkin, *Expectations of care within marriage for older couples.* Australasian Journal on Ageing, 2019. **38**(1): p. E19-E24.
25. de Klerk, M., A. de Boer, and I. Plaisier, *Determinants of informal care-giving in various social relationships in the Netherlands.* Health & Social Care in the Community, 2021. **29**(6): p. 1779-1788.
26. Broese van Groenou, M.I., A. de Boer, and J. Iedema, *Positive and negative evaluation of caregiving among three different types of informal care relationships.* European Journal of Ageing, 2013. **10**(4): p. 301-311.
27. Martire, L.M., *The" relative" efficacy of involving family in psychosocial interventions for chronic illness: are there added benefits to patients and family members?* Families, Systems, & Health, 2005. **23**(3): p. 312.
28. Schulz, R., *The Future of Caregiver Efficacy Research: Commentary on "Long-Term Outcomes of the Benefit-Finding Group Intervention for Alzheimer Family Caregivers".* The American Journal of Geriatric Psychiatry, 2019. **27**(9): p. 995-997.
29. Martire, L.M., et al., *Is it beneficial to involve a family member? A meta-analysis of psychosocial interventions for chronic illness.* Health psychology, 2004. **23**(6): p. 599.
30. Hartmann, M., et al., *Effects of interventions involving the family in the treatment of adult patients with chronic physical diseases: a meta-analysis.* Psychotherapy and psychosomatics, 2010. **79**(3): p. 136-148.
31. Campbell, L.C., et al., *Facilitating research participation and improving quality of life for African American prostate cancer survivors and their intimate partners.* Cancer, 2007. **109**(S2): p. 414-424.
32. Baucom, D.H., et al., *Couple-Based Interventions for Medical Problems.* Behavior Therapy, 2012. **43**(1): p. 61-76.
33. Li, Q. and A.Y. Loke, *A systematic review of spousal couple-based intervention studies for couples coping with cancer: direction for the development of interventions.* Psychooncology, 2014. **23**(7): p. 731-9.
34. Sher, T., et al., *The Partners for Life Program: A Couples Approach to Cardiac Risk Reduction.* Family Process, 2014. **53**(1): p. 131-149.
35. Ratcliff, C.G., et al., *Moving family interventions into the real world: What matters to oncology stakeholders?* Journal of psychosocial oncology, 2019. **37**(2): p. 264-284.
36. Wind, T.R., et al., *The COVID-19 pandemic: The 'black swan'for mental health care and a turning point for e-health.* Internet interventions, 2020. **20**.
37. Semonella, M., et al., *Making a Virtue Out of Necessity: COVID-19 as a Catalyst for Applying Internet-Based Psychological Interventions for Informal Caregivers.* Frontiers in Psychology, 2022: p. 1108.
38. Andersson, G., et al., *Guided Internet-Based CBT for Common Mental Disorders.* Journal of Contemporary Psychotherapy, 2013. **43**(4): p. 223-233.
39. Andersson, G., *Internet interventions: past, present and future.* 2018.
40. Egan, K.J., et al., *Online Training and Support Programs Designed to Improve Mental Health and Reduce Burden Among Caregivers of People With Dementia: A Systematic Review.* Journal of the American Medical Directors Association, 2018. **19**(3): p. 200-206.e1.
41. Sherifali, D., et al., *Impact of Internet-Based Interventions on Caregiver Mental Health: Systematic Review and Meta-Analysis.* J Med Internet Res, 2018. **20**(7): p. e10668.
42. Ploeg, J., et al., *Caregiver-Focused, Web-Based Interventions: Systematic Review and Meta-Analysis (Part 2).* J Med Internet Res, 2018. **20**(10): p. e11247.




For peer review only - http://bmjopen.bmj.com/site/about/guidelines.xhtml

43. Guay, C., et al., *Components and Outcomes of Internet-Based Interventions for Caregivers of Older Adults: Systematic Review.* J Med Internet Res, 2017. **19**(9): p. e313.
44. Shamseer, L., et al., *Preferred reporting items for systematic review and meta-analysis protocols (PRISMA-P) 2015: elaboration and explanation.* Bmj, 2015. **349**.
45. Higgins, J.P., et al., *The Cochrane Collaboration's tool for assessing risk of bias in randomised trials.* Bmj, 2011. **343**.
46. Huang, X., J. Lin, and D. Demner-Fushman. *Evaluation of PICO as a knowledge representation for clinical questions*. in *AMIA annual symposium proceedings*. 2006. American Medical Informatics Association.
47. Tate, C.M. and A. Geliebter, *Intragastric balloon treatment for obesity: review of recent studies.* Advances in therapy, 2017. **34**(8): p. 1859-1875.
48. Dumonceau, J.-M., *Evidence-based review of the Bioenterics intragastric balloon for weight loss.* Obesity surgery, 2008. **18**(12): p. 1611-1617.
49. Hróbjartsson, A., et al., *Assessing risk of bias in randomised clinical trials included in Cochrane Reviews: the why is easy, the how is a challenge.* Cochrane Database of Systematic Reviews, 2013(4).
50. Higgins, J.P. and S.G. Thompson, *Quantifying heterogeneity in a meta-analysis.* Statistics in medicine, 2002. **21**(11): p. 1539-1558.
51. Higgins, J.P., et al., *Measuring inconsistency in meta-analyses.* Bmj, 2003. **327**(7414): p. 557-560.
52. Bucher, H.C., et al., *The results of direct and indirect treatment comparisons in meta-analysis of randomized controlled trials.* Journal of clinical epidemiology, 1997. **50**(6): p. 683-691.
53. O'Toole, M.S., et al., *Cognitive behavioral therapies for informal caregivers of patients with cancer and cancer survivors: a systematic review and meta-analysis.* Psycho-Oncology, 2017. **26**(4): p. 428-437.
54. Gupta, S.K., *Intention-to-treat concept: a review.* Perspectives in clinical research, 2011. **2**(3): p. 109.
55. Guyatt, G.H., et al., *GRADE: an emerging consensus on rating quality of evidence and strength of recommendations.* Bmj, 2008. **336**(7650): p. 924-926.
56. Ryan, R. and S. Hill, *How to GRADE the quality of the evidence.* Cochrane consumers and communication group, 2016. **3**.


For peer review only



For peer review only - http://bmjopen.bmj.com/site/about/guidelines.xhtml

# Applying dyadic digital psychological interventions for reducing caregiver burden in the illness context: A systematic review and a meta-analysis protocol

## PRISMA-P (Preferred Reporting Items for Systematic review and Meta-Analysis Protocols) 2015 checklist: recommended items to address in a systematic review protocol*

| Section and topic | Item No | Checklist item | Location in text (page number) |
|---|---|---|---|
| **ADMINISTRATIVE INFORMATION** | | | |
| Title: | | | |
| Identification | 1a | Identify the report as a protocol of a systematic review | 1 |
| Update | 1b | If the protocol is for an update of a previous systematic review, identify as such | N/A |
| Registration | 2 | If registered, provide the name of the registry (such as PROSPERO) and registration number | 7 |
| Authors: | | | |
| Contact | 3a | Provide name, institutional affiliation, e-mail address of all protocol authors; provide physical mailing address of corresponding author | 1 |
| Contributions | 3b | Describe contributions of protocol authors and identify the guarantor of the review | 11 |
| Amendments | 4 | If the protocol represents an amendment of a previously completed or published protocol, identify as such and list changes; otherwise, state plan for documenting important protocol amendments | 7 |
| Support: | | | |
| Sources | 5a | Indicate sources of financial or other support for the review | 11 |
| Sponsor | 5b | Provide name for the review funder and/or sponsor | 11 |
| Role of sponsor or funder | 5c | Describe roles of funder(s), sponsor(s), and/or institution(s), if any, in developing the protocol | 11 |
| **INTRODUCTION** | | | |
| Rationale | 6 | Describe the rationale for the review in the context of what is already known | 4-6 |
| Objectives | 7 | Provide an explicit statement of the question(s) the review will address with reference to participants, interventions, comparators, and outcomes (PICO) | 6 |

For peer review only

| | | | |
|---|---|---|---|
| **METHODS** | | | |
| Eligibility criteria | 8 | Specify the study characteristics (such as PICO, study design, setting, time frame) and report characteristics (such as years considered, language, publication status) to be used as criteria for eligibility for the review | 7-8 |
| Information sources | 9 | Describe all intended information sources (such as electronic databases, contact with study authors, trial registers or other grey literature sources) with planned dates of coverage | 8 |
| Search strategy | 10 | Present draft of search strategy to be used for at least one electronic database, including planned limits, such that it could be repeated | 9 |
| Study records: | | | |
| Data management | 11a | Describe the mechanism(s) that will be used to manage records and data throughout the review | 9 |
| Selection process | 11b | State the process that will be used for selecting studies (such as two independent reviewers) through each phase of the review (that is, screening, eligibility and inclusion in meta-analysis) | 9 |
| Data collection process | 11c | Describe planned method of extracting data from reports (such as piloting forms, done independently, in duplicate), any processes for obtaining and confirming data from investigators | 9 |
| Data items | 12 | List and define all variables for which data will be sought (such as PICO items, funding sources), any pre-planned data assumptions and simplifications | 9 |
| Outcomes and prioritization | 13 | List and define all outcomes for which data will be sought, including prioritization of main and additional outcomes, with rationale | 9-10 |
| Risk of bias in individual studies | 14 | Describe anticipated methods for assessing risk of bias of individual studies, including whether this will be done at the outcome or study level, or both; state how this information will be used in data synthesis | 10 |
| Data synthesis | 15a | Describe criteria under which study data will be quantitatively synthesised | N/A |
| | 15b | If data are appropriate for quantitative synthesis, describe planned summary measures, methods of handling data and methods of combining data from studies, including any planned exploration of consistency (such as $I^2$, Kendall's $\tau$) | 11 |
| | 15c | Describe any proposed additional analyses (such as sensitivity or subgroup analyses, meta-regression) | N/A |
| | 15d | If quantitative synthesis is not appropriate, describe the type of summary planned | |
| Meta-bias(es) | 16 | Specify any planned assessment of meta-bias(es) (such as publication bias across studies, selective reporting within studies) | 10-11 |
| Confidence in cumulative evidence | 17 | Describe how the strength of the body of evidence will be assessed (such as GRADE) | 11 |

N/A= Not Applicable

*** It is strongly recommended that this checklist be read in conjunction with the PRISMA-P Explanation and Elaboration (cite when available) for important clarification on the items. Amendments to a review protocol should be tracked and dated.** **The copyright for PRISMA-P (including checklist) is held by the PRISMA-P Group and is distributed under a Creative Commons Attribution Licence 4.0.**

For peer review only

*From: Shamseer L, Moher D, Clarke M, Ghersi D, Liberati A, Petticrew M, Shekelle P, Stewart L, PRISMA-P Group. Preferred reporting items for systematic review and meta-analysis protocols (PRISMA-P) 2015: elaboration and explanation. BMJ. 2015 Jan 2;349(jan02 1):g7647.*

For peer review only

BMJ Open

# Applying dyadic digital psychological interventions for reducing caregiver burden in the illness context: A systematic review and a meta-analysis protocol



| | |
|---|---|
| Journal: | *BMJ Open* |
| Manuscript ID | bmjopen-2022-070279.R1 |
| Article Type: | Protocol |
| Date Submitted by the Author: | 03-Mar-2023 |



| | |
|---|---|
| Complete List of Authors: | Semonella, Michelle; Bar-Ilan University, Department of Psychology, Bar-Ilan University 5290002, Israel<br>Bertuzzi, Vanessa; Università Cattolica del Sacro Cuore, Psicologia<br>Dekel, Rachel; Bar-Ilan University, School of Social Work<br>Andersson, Gerhard; Linkoping University,<br>Pietrabissa, Giada; IRCCS Istituto Auxologico Italiano, Ospedale San Giuseppe, Psychology Research Laboratory; Universita Cattolica del Sacro Cuore, Department of Psychology<br>Vilchinsky, Noa; Universitat Bar-Ilan, Department of Psychology |



| | |
|---|---|
| <b>Primary Subject Heading</b>: | Mental health |
| Secondary Subject Heading: | Research methods, Palliative care |
| Keywords: | MENTAL HEALTH, Quality in health care < HEALTH SERVICES ADMINISTRATION & MANAGEMENT, Protocols & guidelines < HEALTH SERVICES ADMINISTRATION & MANAGEMENT |


SCHOLARONE™
Manuscripts

# Applying dyadic digital psychological interventions for reducing caregiver burden in the illness context: A systematic review and a meta-analysis protocol

Michelle Semonella[1]; Vanessa Bertuzzi[2]; Rachel Dekel[3]; Gerhard Andersson[4,5,6]; Giada Pietrabissa[2,7]; Noa Vilchinsky[1]

[1]Department of Psychology, Bar-Ilan University, Ramat Gan, Israel
[2]Department of Psychology, Catholic University of Milan, Milan, Italy
[3]School of Social Work, Bar-Ilan University, Ramat Gan, Israel
[4]Department of Behavioral Sciences and Learning, Linköping University, Linköping, Sweden
[5]Department of Biomedical and Clinical Sciences, Linköping University, Linköping, Sweden
[6]Department of Clinical Neuroscience, Karolinska Institute, Stockholm, Sweden
[7] Psychology Research Laboratory, IRCCS Istituto Auxologico Italiano, Milan, Italy

Corresponding Author: Michelle Semonella; michelle.semonella@biu.ac.il

Keywords: informal caregivers; digital psychological interventions; caregiver burden; dyadic interventions; eHealth; systematic review; meta-analysis



For peer review only - http://bmjopen.bmj.com/site/about/guidelines.xhtml

## *Abstract*

**Introduction:** Providing informal care to one's romantic partner who is ill may become a highly distressing and demanding task. Based on the innovative dyadic coping model, several support interventions have been developed to alleviate informal caregivers' burden, including both caregivers' and care receivers' needs. Considering the unique challenges characterizing the caregiving phenomenon, such as geographical barriers and time restrictions, digital solutions should be considered. However, there is a lack of research examining the effectiveness of dyadic digital solutions. Thus, this review aims to examine the existing literature on the efficacy of dyadic digital psychological interventions designed for caregivers and their care-receivers couples within the illness context.

**Methods and analysis:** Randomized controlled trials targeting caregivers' burden among dyads of informal caregivers and care-receivers will be identified via an electronic search of the following databases: PubMed, Embase, the Cochrane Library, Cinhal, Scopus, PsycINFO, MEDLINE, and supplemented by hand searching of previous systematic reviews. The search will be undertaken following the PICO elements. If possible, a meta-analysis will be conducted to examine: 1. The effectiveness of dyadic digital psychological interventions for reducing caregivers' burden (primary outcome) among caregivers who are in a romantic relationship with the care receivers; 2. The effectiveness of dyadic digital psychological interventions on secondary outcomes such as anxiety, depression, stress, quality of life, well-being, and self-efficacy among caregivers and care-receivers; 3. Moderating effects of clinical and methodological factors on caregivers' burden. Prior to inclusion in the review, retrieved papers will be critically appraised by two independent reviewers. The Cochrane Risk of Bias tool will assess the risk of bias for randomized controlled trials.




For peer review only - http://bmjopen.bmj.com/site/about/guidelines.xhtml

**Ethics and dissemination:** Ethical approval is not required as no primary data will be collected. Findings will be disseminated through peer-reviewed publications, presentations at academic conferences, and lay summaries for various stakeholders.

**PROSPERO registration number** CRD42022299125

For peer review only



For peer review only - http://bmjopen.bmj.com/site/about/guidelines.xhtml

Strengths and limitations of this study

- **To increase the quality of included studies and reduce methodological heterogeneity, only studies assessed as having a low or moderate risk of bias, according to the Cochrane Collaboration's Risk of Bias tool 2.0, will be included.**
- **Following the Centre and Reviews and Dissemination guidance, as well as the PRISMA-P guidelines, this protocol adopts independent study selection, independent data extraction, and independent risk of bias assessment by two reviewers.**
- **Only randomized controlled trials will be included to maintain a high methodological standard.**
- **The studies may not have collected all the variables of interest. Thus, we might not be able to examine all candidate-moderators.**
- **The review will be restricted to studies published in English, which might be indicative of a language bias.**



For peer review only - http://bmjopen.bmj.com/site/about/guidelines.xhtml

# INTRODUCTION

## *Informal caregiving*

Informal care is defined as the unpaid care provided to an older, frail, or ill person, by a person such as a spouse, parent, child, another relative, neighbor, friend, or other non-kin [1]. The phenomenon of providing informal care is growing, mostly due to the growth in the aging population, the increasing prevalence of chronic illnesses, and more effective medical treatments saving lives. To date, it is estimated that 44 million people in Europe provide informal care to family or friends [2]. Indeed, In Europe, 80% of Long-Term Care (LCT) for non-independent people is provided by informal caregivers; this estimation makes them an essential element of the care system in Europe [3]. At the same time, the growing number of caregivers has made caregiving a public health issue [4, 5]. Being an informal caregiver can be a stressful experience and is often associated with negative psychosocial and health consequences as well as financial strains, all of which could increase caregiving burden [6, 7]. Caregiver burden is a multidimensional concept consisting of physical, psychological, emotional, social, and financial stressors linked with the caregiving experience which might contribute to caregivers' physical and psychological illnesses [8-10]. Indeed, due to their extensive caregiving responsibilities, many caregivers report physical exhaustion, and even negative health consequences such as increased cardiovascular reactivity and poor immune

For peer review only



For peer review only - http://bmjopen.bmj.com/site/about/guidelines.xhtml

system response [11, 12]. It is therefore necessary to find ways to alleviate caregivers' burden and distress by developing suitable and feasible psychological support interventions or implementing existing ones. When designing or implementing interventions it is important to take into account the characteristics, limitations, and features that characterizes informal care, such as its dyadic nature and caregivers' difficulties in terms of lack of time and money.

***Novel approaches to interventions for caregivers: the call for dyadic and digital solutions***

During the last twenty years, there has been a shift in perspective in the health psychology literature: from an individual perspective of coping with the illness to a dyadic one [13-15]. Researchers have demonstrated that both members of the dyad actively share resources and manage stressors together as a couple. Specifically, a new conceptualization has emerged viewing illness as a “we-disease” [16], which taps mutual influences between two coping partners in a dyad [17-19]. Particularly, providing care in romantic relationships differs from other kinds of relationships for three main reasons. First, romantic partners are most likely to live together with the care recipients, and therefore usually provide support when needed. Second, caregivers who are in a romantic relationship are usually older than adult children who provide care for their parents, thus they may have their own health problems which may lead to greater perceived stress while providing informal care. Third, the romantic partner is usually the most important attachment figure for the care receiver [20], and many expectations



For peer review only - http://bmjopen.bmj.com/site/about/guidelines.xhtml

are directed toward him/her on behalf of the patient [21, 22]. Thus, caregiving spouses in particular might be at higher risk for caregiver burden [23]. Thus, given that caregiving is conceptualized as a dyadic process rather than an individualized one [13], any intervention developed for caregivers should be designed dyadically, taking into account both caregivers' and care receivers' needs and consequences, as well as the relationship between the two. Indeed, different dyadic interventions have been developed to ease caregivers' stress and help them better cope with their condition in various health contexts; and they are effective in improving outcomes (such as caregiver burden, quality of life, stress, anxiety, depression) in both informal caregivers and care-receivers [24-27].

Although dyadic interventions were shown to be successful and beneficial for both members of the dyad [28-31], most of them have been delivered in person, requiring the participation of both members which may limit their ultimate scalability and accessibility [32]. It is, therefore, necessary to develop interventions that are dyadic in nature – involving both partners – while at the same time being feasible. In recent years using information technology has become a regular part of our daily life, and also common in the clinical field and health care. During the coronavirus pandemic, psychological support delivered through digital solutions became more relevant, accepted, and feasible [33]. Indeed, the Covid-19 outbreak facilitated the acceptability and willingness to use information technology tools to deliver psychological

For peer review only

interventions [34]. Thanks to psychologically-based digital interventions, it is possible to reach patients from a distance and research suggests that this mode of delivery can be as effective as other treatment formats [35], and serve as a complement or even alternative to regular face-to-face treatments [36]. Digital interventions would be particularly useful and efficient to fill gaps due to caregiving conditions, as they suit caregivers' needs in terms of lack of time, money, and distance [34]. Moreover, digital solutions can have beneficial effects on caregivers' mental health, including reduction of depression, stress, anxiety, and caregiver burden [37, 38], and have also been found to increase self-efficacy, self-esteem, and strain of caregivers of adults with chronic conditions [39] as well as enhance the sense of competence, coping skills and strategies, and quality of life in caregivers of older adults [40]. A recently published systematic review on dyadic psychological eHealth interventions demonstrated that by integrating the dyadic approach with technology, interventions were found most suitable to meet caregivers' needs, as well as feasible and cost-effective [41]. The current review aims to add more accurate knowledge on the subject by considering dyadic romantic partner relationships only; focusing on caregiver burden as the prespecified primary endpoint, and conducting a meta-analysis limited to randomized controlled trials only.

**Aim of the study**



For peer review only - http://bmjopen.bmj.com/site/about/guidelines.xhtml

To the best of our knowledge, no review or meta-analysis has systematically evaluated the current research on dyadic digital psychological interventions for informal caregivers. This study aims to systematically review the existing literature on the efficacy of dyadic digital psychological interventions designed for couples of informal caregivers and their care-receivers within the illness context.

Specifically, we aim to examine:

1. The efficacy of dyadic digital psychological interventions for reducing caregivers' burden among informal caregivers who are in a romantic relationship with adults with a chronic illness;
2. The effectiveness of dyadic digital psychological interventions on secondary outcomes such as anxiety, depression, stress, quality of life, well-being, self-efficacy, and relationship satisfaction among informal caregivers and care-receivers;
3. The moderating effects of clinical and methodological factors on caregivers' burden.

## METHODS AND ANALYSIS

This protocol adopts the Preferred Items for Reporting of Systematic Reviews and Meta-Analyses Protocol (PRISMA) guidelines [42] (online supplement appendix 1) and the Cochrane Risk of Bias tool to assess the risk of bias for randomized controlled trials [43].

### Study registration



For peer review only - http://bmjopen.bmj.com/site/about/guidelines.xhtml

Following the PRISMA guidelines [42], our systematic review protocol was registered with the International Prospective Register of Systematic Reviews (PROSPERO) on March 3rd, 2022 (registration number CRD42022299125). Any important protocol amendments will be recorded in PROSPERO and published with the results of the review.

## Eligibility criteria

Inclusion and exclusion criteria are categorized by population, interventions, comparators, outcomes, and study design (PICOS) [44].

Eligible studies for inclusion will be in English language peer-reviewed studies or unpublished studies such as doctoral thesis to reduce publication bias.

## Population

We will include studies examining adults (18 years or older) who provide informal care and their care-receivers in a romantic relationship. Thus, we will not include studies that include formal caregivers; studies in which the sample included caregiving parents, siblings, or other non-romantic relationships; and finally, studies that do not collect data on both informal caregivers and care-receivers. No restrictions have been placed on participants' gender and ethnicity.

## Interventions

Any psychological, psychoeducational, psychosocial, or combination of psychological interventions using specific therapeutic principles and techniques, delivered via a technological tool (e.g., computer, telephone, videos). Any other type of interventions such as



For peer review only - http://bmjopen.bmj.com/site/about/guidelines.xhtml

pharmacological interventions, acupuncture, smoking cessation, etc. will be excluded. All the interventions included must be dyadic interventions addressing, targeting, and providing data on both informal caregivers and care-receivers. Individual intervention or studies that provide data on either caregiver or care-receiver only will not be included in the current review.

**Comparator**

Both active and inactive comparators will be eligible, such as no intervention, usual care, waiting list,

and attention control.

**Outcomes**

Primary outcomes

Studies will be included only if one or more standardized measurements of caregivers' burden have been used.

Secondary outcomes

Secondary outcomes of interest are quantitative measurements listed below:

- Anxiety;
- Depression;
- Stress;
- Quality of life;
- Well-being;
- Self-efficacy.



For peer review only - http://bmjopen.bmj.com/site/about/guidelines.xhtml

## Study design

We will include only randomized controlled trials, to optimize the internal validity of this review.

## Information sources

## Search strategy

Two reviewers (MS and VB) will independently search the following database for relevant articles: PubMed, Embase, the Cochrane Library, Cinhal, Scopus, PsycINFO, and MEDLINE. For each database, a comprehensive search strategy will be developed under the PICO (Population, Intervention, Comparison, and Outcome) framework [44]. Boolean and truncation operators will be used to systematically combine search terms and to screen for a list of pre-specified search terms in titles and abstracts. (online supplement appendix 2).

No restrictions will be placed on the publication period. The reference lists of all selected articles and relevant systematic review will be manually screened to identify further relevant records for possible inclusion [45, 46]

Additionally, studies from grey literature (e.g., thesis dissertation, conference papers/abstract) will be searched in OpenGrey (http://www. Open grey. eu/) and will be included in case they fulfill the inclusion criteria.

Data will be exported to Endnote X8 (Clarivate Analytics, Philadelphia, Pennsylvania, USA) and Excel for the title and abstract screening.

## Study records

Data management



For peer review only - http://bmjopen.bmj.com/site/about/guidelines.xhtml

Data will be imported into Endnote X8 (Clarivate Analytics, Philadelphia, Pennsylvania, USA) and duplicate records will be removed, while (if possible), Stata V.16.0 (StataCorp) will be used for meta-analysis.

Selection process

Two reviewers will independently screen study titles and abstracts retrieved from searches; they will perform full paper checks of identified potentially eligible studies. The PICOS criteria will be used to select eligible studies. In the results manuscript will be documented and reported the overall reasons for exclusion at the full-text screening, using a PRISMA flow chart. A detailed overview of the reasons for the inclusion/exclusion of studies at the PICOS item level will be presented in a table in the results manuscript. Disagreements between reviewers will be solved by consulting a third reviewer. The overall selection process will be represented as a PRIMA flow diagram.

**Data extraction**

All reviewers will be involved in reaching a consensus on the data to be extracted and the terminology used before the start of the screening. Data extraction will be conducted independently by two reviewers (MS and VB). One reviewer (MS) will screen the titles of each study based on the eligibility criteria and when these are deemed relevant, she will go through abstracts. Once narrowed down by abstracts, a full-text review process will be completed in duplicate by two reviewers (MS and VB) for studies that met the eligibility criteria at screening and for studies with unclear relevance. If there are any disagreements, a third independent reviewer (either NV or RD) will be consulted to resolve discrepancies. Original authors of studies identified will be contacted if the full-text paper was not available or the relevance of a paper was unclear. We will assess the inter-rater agreement by kappa statistic using



For peer review only - http://bmjopen.bmj.com/site/about/guidelines.xhtml

GraphPad Software. A kappa value of 0.61–0.80 reflects substantial agreement , and a kappa-value of 0.81–1.00 reflects (almost) perfect agreement [47].

**Risk of bias**

The Cochrane Collaboration's Risk for Bias tool will be used to evaluate the risk of bias and the quality of the studies in the form of randomized controlled trial [43]. Thanks to this tool each domain of potential bias will be classified as "low risk" or "unclear risk" or "high risk" [48].

Two researchers will work independently. Any disagreement will be solved by a third reviewer.

**Data synthesis**

A narrative synthesis will be undertaken to present and explain the findings. Two reviewers (MS, VB) will independently read the selected studies and will propose an initial framework for its synthesis. These frameworks will serve as a basis for a consensus synthesis framework, decided by all reviewers in a group discussion. All reviewers will be involved in writing the narrative synthesis, following a joint iterative process of organizing and cross-validating the reported results.

Meta-analysis

If data allow, a meta-analysis will be performed to explore the effectiveness of dyadic digital psychological interventions for reducing caregivers' burden among informal caregivers by calculating post-treatment between-group standardized mean effect sizes for the primary outcome using Hedges' g. In addition, the effectiveness of dyadic digital psychological interventions will be examined on selected secondary outcomes (e.g., depression, anxiety,



For peer review only - http://bmjopen.bmj.com/site/about/guidelines.xhtml

stress, quality of life, well-being, and self-efficacy) among both informal caregivers and/or care-receivers.

Dichotomous outcomes will be measured by risk ratio (RR) and its 95% confidence interval. Continuous outcomes will be measured by calculating the mean difference (MD) with 95% CI when the studies use the same instrument for assessing the outcome. The standardized mean difference will be used when studies use different instruments.

Assessment of heterogeneity

A random-effect model will be performed for all the analyses because of the potential heterogeneity among clinical trial results. The heterogeneity between-study will be measured using the Cochrane's test of heterogeneity (Q) and will be reported using $I^2$ statistics, alongside CIs [49, 50]. The indirect comparisons between technology will be assessed using the Bucher and Glenny method [51].

Moderator analyses

Prespecified subgroup analyses will be performed to assess the potential association of some clinical factors with the primary outcome. Subgroups were defined according to toa. Study quality characteristics: control type (active VS non-active); b. Informal caregivers characteristics: age, women percentage, and education; c. Patient characteristics: age, women percentage, education, type of illness; d. Intervention characteristics: number of sessions, duration, type of therapy (e.g., CBT VS mindfulness), modality (tool used to deliver the intervention (e.g., telephone VS video) [52].

Dealing with missing data



For peer review only - http://bmjopen.bmj.com/site/about/guidelines.xhtml

Intention-to-treat data will be used when possible [53].

## Confidence in cumulative evidence

The Grading of Recommendations Assessment, Development, and Evaluation (GRADE) [54, 55] tool will be used to assess the certainty of the evidence of both primary outcome and secondary outcomes as “high”, “moderate”, “low” or “very low”, according to the GRADE approach. A summary of evidence will be presented in a table.

## Patient and public involvement

There was no patient and public involvement in the development of this protocol.

## Ethics and dissemination

Any identifiable patient data will be excluded; thus, ethical approval and participant consent are not required. Findings will be disseminated through peer-reviewed publications, presentations at academic conferences, and lay summaries for various stakeholders.

## Contributors

MS contributed to the conception and design of the study and wrote the manuscript. NV contributed to the conception and design of the study and critically revised the manuscript drafts. VB, GA, RD, and GP critically revised the study design, assisted in data synthesis and analysis, and revised the manuscript’s final draft. All authors approved the final manuscript.

## Funding

This work is funded by the European Union’s Horizon 2020 research and innovation program under the Marie-Sklodowska Curie grant agreement no 814072.

## Competing interests



For peer review only - http://bmjopen.bmj.com/site/about/guidelines.xhtml

None declared.

For peer review only



For peer review only - http://bmjopen.bmj.com/site/about/guidelines.xhtml

## References

1. Triantafillou, J., et al., *Informal care in the long-term care system European overview paper.* 2010.
2. de Lanchy, G., N. Lorentz, and K. Leduc, *Long term care workforce: Employment and working conditions-Luxembourg*, in *Long term care workforce: Employment and working conditions*. 2020, Publications Office of the European Commission.
3. Naiditch, M., et al., *User perspectives in long-term care and the role of informal carers*, in *Long-Term Care in Europe*. 2013, Springer. p. 45-80.
4. Schulz, R. and T.L. Patterson, *Caregiving in geriatric psychiatry.* The American Journal of Geriatric Psychiatry, 2004. **12**(3): p. 234-237.
5. Edwards, V.J., et al., *Characteristics and Health Status of Informal Unpaid Caregivers - 44 States, District of Columbia, and Puerto Rico, 2015-2017.* MMWR. Morbidity and mortality weekly report, 2020. **69**(7): p. 183-188.
6. Hwang, B., et al., *Caregiving for patients with heart failure: impact on patients' families.* Am J Crit Care, 2011. **20**(6): p. 431-41; quiz 442.
7. Metzelthin, S.F., et al., *Positive and negative outcomes of informal caregiving at home and in institutionalised long-term care: a cross-sectional study.* BMC geriatrics, 2017. **17**(1): p. 232.
8. Jones, C., *Careers in project networks: The case of the film industry.* The boundaryless career: A new employment principle for a new organizational era, 1996. **58**: p. 58-75.
9. Li, Q.P., Y.W. Mak, and A.Y. Loke, *Spouses' experience of caregiving for cancer patients: a literature review.* Int Nurs Rev, 2013. **60**(2): p. 178-87.
10. Pottie, C.G., et al., *Informal caregiving of hospice patients.* J Palliat Med, 2014. **17**(7): p. 845-56.
11. Choi, S. and J. Seo, *Analysis of caregiver burden in palliative care: An integrated review.* Nursing Forum, 2019. **54**(2): p. 280-290.
12. Pop, R.S., A. Puia, and D. Mosoiu, *Factors influencing the quality of life of the primary caregiver of a palliative patient: narrative review.* Journal of palliative medicine, 2022. **25**(5): p. 813-829.
13. Revenson, T.A., et al., *Caregiving as a dyadic process*, in *Caregiving in the illness context*. 2016, Springer. p. 25-37.
14. Gouin, J.-P., et al., *Dyadic coping and inflammation in the context of chronic stress.* Health Psychology, 2016. **35**(10): p. 1081.



For peer review only - http://bmjopen.bmj.com/site/about/guidelines.xhtml

15. Carr, R., et al., *Dyadic interventions to promote physical activity and reduce sedentary behaviour: Systematic review and meta-analysis.* Health Psychology Review, 2019. **13**(1): p. 91-109.

16. Kayser, K., L.E. Watson, and J.T. Andrade, *Cancer as a" we-disease": Examining the process of coping from a relational perspective.* Families, Systems, & Health, 2007. **25**(4): p. 404.

17. Acitelli, L.K. and H.J. Badr, *My Illness or Our Illness? Attending to the Relationship When One Partner Is Ill*, in *Couples coping with stress: Emerging perspectives on dyadic coping.* 2005, American Psychological Association: Washington, DC, US. p. 121-136.

18. Rohrbaugh, M.J., et al., *Prognostic significance of spouse we talk in couples coping with heart failure.* Journal of Consulting and Clinical Psychology, 2008. **76**(5): p. 781-789.

19. Revenson, T.A., et al., *Interventions to support caregivers*, in *Caregiving in the illness context.* 2016, Springer. p. 90-104.

20. Pinquart, M. and S. Sörensen, *Spouses, adult children, and children-in-law as caregivers of older adults: a meta-analytic comparison.* Psychology and aging, 2011. **26**(1): p. 1.

21. Cash, B., J. Warburton, and S. Hodgkin, *Expectations of care within marriage for older couples.* Australasian Journal on Ageing, 2019. **38**(1): p. E19-E24.

22. de Klerk, M., A. de Boer, and I. Plaisier, *Determinants of informal care-giving in various social relationships in the Netherlands.* Health & Social Care in the Community, 2021. **29**(6): p. 1779-1788.

23. Broese van Groenou, M.I., A. de Boer, and J. Iedema, *Positive and negative evaluation of caregiving among three different types of informal care relationships.* European Journal of Ageing, 2013. **10**(4): p. 301-311.

24. Martire, L.M., *The" relative" efficacy of involving family in psychosocial interventions for chronic illness: are there added benefits to patients and family members?* Families, Systems, & Health, 2005. **23**(3): p. 312.

25. Schulz, R., *The Future of Caregiver Efficacy Research: Commentary on "Long-Term Outcomes of the Benefit-Finding Group Intervention for Alzheimer Family Caregivers".* The American Journal of Geriatric Psychiatry, 2019. **27**(9): p. 995-997.

26. Martire, L.M., et al., *Is it beneficial to involve a family member? A meta-analysis of psychosocial interventions for chronic illness.* Health psychology, 2004. **23**(6): p. 599.



For peer review only - http://bmjopen.bmj.com/site/about/guidelines.xhtml

27. Hartmann, M., et al., *Effects of interventions involving the family in the treatment of adult patients with chronic physical diseases: a meta-analysis.* Psychotherapy and psychosomatics, 2010. **79**(3): p. 136-148.

28. Pucciarelli, G., et al., *Effectiveness of dyadic interventions to improve stroke patient–caregiver dyads' outcomes after discharge: A systematic review and meta-analysis study.* European Journal of Cardiovascular Nursing, 2020. **20**(1): p. 14-33.

29. Bannon, S.M., et al., *Emotional distress in neuro-ICU survivor–caregiver dyads: The recovering together randomized clinical trial.* Health Psychology, 2022. **41**: p. 268-277.

30. Sher, T., et al., *The Partners for Life Program: A Couples Approach to Cardiac Risk Reduction.* Family Process, 2014. **53**(1): p. 131-149.

31. Campbell, L.C., et al., *Facilitating research participation and improving quality of life for African American prostate cancer survivors and their intimate partners.* Cancer, 2007. **109**(S2): p. 414-424.

32. Ratcliff, C.G., et al., *Moving family interventions into the real world: What matters to oncology stakeholders?* Journal of psychosocial oncology, 2019. **37**(2): p. 264-284.

33. Wind, T.R., et al., *The COVID-19 pandemic: The 'black swan'for mental health care and a turning point for e-health.* Internet interventions, 2020. **20**.

34. Semonella, M., et al., *Making a Virtue Out of Necessity: COVID-19 as a Catalyst for Applying Internet-Based Psychological Interventions for Informal Caregivers.* Frontiers in Psychology, 2022: p. 1108.

35. Andersson, G., et al., *Guided Internet-Based CBT for Common Mental Disorders.* Journal of Contemporary Psychotherapy, 2013. **43**(4): p. 223-233.

36. Andersson, G., *Internet interventions: past, present and future.* 2018.

37. Egan, K.J., et al., *Online Training and Support Programs Designed to Improve Mental Health and Reduce Burden Among Caregivers of People With Dementia: A Systematic Review.* Journal of the American Medical Directors Association, 2018. **19**(3): p. 200-206.e1.

38. Sherifali, D., et al., *Impact of Internet-Based Interventions on Caregiver Mental Health: Systematic Review and Meta-Analysis.* J Med Internet Res, 2018. **20**(7): p. e10668.

39. Ploeg, J., et al., *Caregiver-Focused, Web-Based Interventions: Systematic Review and Meta-Analysis (Part 2).* J Med Internet Res, 2018. **20**(10): p. e11247.

40. Guay, C., et al., *Components and Outcomes of Internet-Based Interventions for Caregivers of Older Adults: Systematic Review.* J Med Internet Res, 2017. **19**(9): p. e313.

41. Shaffer, K.M., et al., *Dyadic Psychosocial eHealth Interventions: Systematic Scoping Review.* J Med Internet Res, 2020. **22**(3): p. e15509.



For peer review only - http://bmjopen.bmj.com/site/about/guidelines.xhtml

42. Shamseer, L., et al., *Preferred reporting items for systematic review and meta-analysis protocols (PRISMA-P) 2015: elaboration and explanation.* Bmj, 2015. **349**.

43. Higgins, J.P., et al., *The Cochrane Collaboration's tool for assessing risk of bias in randomised trials.* Bmj, 2011. **343**.

44. Huang, X., J. Lin, and D. Demner-Fushman. *Evaluation of PICO as a knowledge representation for clinical questions*. in *AMIA annual symposium proceedings*. 2006. American Medical Informatics Association.

45. Tate, C.M. and A. Geliebter, *Intragastric balloon treatment for obesity: review of recent studies.* Advances in therapy, 2017. **34**(8): p. 1859-1875.

46. Dumonceau, J.-M., *Evidence-based review of the Bioenterics intragastric balloon for weight loss.* Obesity surgery, 2008. **18**(12): p. 1611-1617.

47. Viera, A.J. and J.M. Garrett, *Understanding interobserver agreement: the kappa statistic.* Fam med, 2005. **37**(5): p. 360-363.

48. Hróbjartsson, A., et al., *Assessing risk of bias in randomised clinical trials included in Cochrane Reviews: the why is easy, the how is a challenge.* Cochrane Database of Systematic Reviews, 2013(4).

49. Higgins, J.P. and S.G. Thompson, *Quantifying heterogeneity in a meta-analysis.* Statistics in medicine, 2002. **21**(11): p. 1539-1558.

50. Higgins, J.P., et al., *Measuring inconsistency in meta-analyses.* Bmj, 2003. **327**(7414): p. 557-560.

51. Bucher, H.C., et al., *The results of direct and indirect treatment comparisons in meta-analysis of randomized controlled trials.* Journal of clinical epidemiology, 1997. **50**(6): p. 683-691.

52. O'Toole, M.S., et al., *Cognitive behavioral therapies for informal caregivers of patients with cancer and cancer survivors: a systematic review and meta-analysis.* Psycho-Oncology, 2017. **26**(4): p. 428-437.

53. Gupta, S.K., *Intention-to-treat concept: a review.* Perspectives in clinical research, 2011. **2**(3): p. 109.

54. Guyatt, G.H., et al., *GRADE: an emerging consensus on rating quality of evidence and strength of recommendations.* Bmj, 2008. **336**(7650): p. 924-926.

55. Ryan, R. and S. Hill, *How to GRADE the quality of the evidence.* Cochrane consumers and communication group, 2016. **3**.


For peer review only

# Applying dyadic digital psychological interventions for reducing caregiver burden in the illness context: A systematic review and a meta-analysis protocol

## PRISMA-P (Preferred Reporting Items for Systematic review and Meta-Analysis Protocols) 2015 checklist: recommended items to address in a systematic review protocol*

For peer review only

| Section and topic | Item No | Checklist item | Location in text (page number) |
|---|---|---|---|
| **ADMINISTRATIVE INFORMATION** | | | |
| Title: | | | |
| Identification | 1a | Identify the report as a protocol of a systematic review | 1 |
| Update | 1b | If the protocol is for an update of a previous systematic review, identify as such | N/A |
| Registration | 2 | If registered, provide the name of the registry (such as PROSPERO) and registration number | 7 |
| Authors: | | | |
| Contact | 3a | Provide name, institutional affiliation, e-mail address of all protocol authors; provide physical mailing address of corresponding author | 1 |
| Contributions | 3b | Describe contributions of protocol authors and identify the guarantor of the review | 11 |
| Amendments | 4 | If the protocol represents an amendment of a previously completed or published protocol, identify as such and list changes; otherwise, state plan for documenting important protocol amendments | 7 |
| Support: | | | |
| Sources | 5a | Indicate sources of financial or other support for the review | 11 |
| Sponsor | 5b | Provide name for the review funder and/or sponsor | 11 |
| Role of sponsor or funder | 5c | Describe roles of funder(s), sponsor(s), and/or institution(s), if any, in developing the protocol | 11 |
| **INTRODUCTION** | | | |
| Rationale | 6 | Describe the rationale for the review in the context of what is already known | 4-6 |
| Objectives | 7 | Provide an explicit statement of the question(s) the review will address with reference to participants, interventions, comparators, and outcomes (PICO) | 6 |

| | | | |
|---|---|---|---|
| **METHODS** | | | |
| Eligibility criteria | 8 | Specify the study characteristics (such as PICO, study design, setting, time frame) and report characteristics (such as years considered, language, publication status) to be used as criteria for eligibility for the review | 7-8 |
| Information sources | 9 | Describe all intended information sources (such as electronic databases, contact with study authors, trial registers or other grey literature sources) with planned dates of coverage | 8 |
| Search strategy | 10 | Present draft of search strategy to be used for at least one electronic database, including planned limits, such that it could be repeated | 9 |
| Study records: | | | |
| Data management | 11a | Describe the mechanism(s) that will be used to manage records and data throughout the review | 9 |
| Selection process | 11b | State the process that will be used for selecting studies (such as two independent reviewers) through each phase of the review (that is, screening, eligibility and inclusion in meta-analysis) | 9 |
| Data collection process | 11c | Describe planned method of extracting data from reports (such as piloting forms, done independently, in duplicate), any processes for obtaining and confirming data from investigators | 9 |
| Data items | 12 | List and define all variables for which data will be sought (such as PICO items, funding sources), any pre-planned data assumptions and simplifications | 9 |
| Outcomes and prioritization | 13 | List and define all outcomes for which data will be sought, including prioritization of main and additional outcomes, with rationale | 9-10 |
| Risk of bias in individual studies | 14 | Describe anticipated methods for assessing risk of bias of individual studies, including whether this will be done at the outcome or study level, or both; state how this information will be used in data synthesis | 10 |
| Data synthesis | 15a | Describe criteria under which study data will be quantitatively synthesised | N/A |
| | 15b | If data are appropriate for quantitative synthesis, describe planned summary measures, methods of handling data and methods of combining data from studies, including any planned exploration of consistency (such as $I^2$, Kendall's $\tau$) | 11 |
| | 15c | Describe any proposed additional analyses (such as sensitivity or subgroup analyses, meta-regression) | N/A |
| | 15d | If quantitative synthesis is not appropriate, describe the type of summary planned | |
| Meta-bias(es) | 16 | Specify any planned assessment of meta-bias(es) (such as publication bias across studies, selective reporting within studies) | 10-11 |
| Confidence in cumulative evidence | 17 | Describe how the strength of the body of evidence will be assessed (such as GRADE) | 11 |

N/A= Not Applicable

*** It is strongly recommended that this checklist be read in conjunction with the PRISMA-P Explanation and Elaboration (cite when available) for important clarification on the items. Amendments to a review protocol should be tracked and dated.** **The copyright for PRISMA-P (including checklist) is held by the PRISMA-P Group and is distributed under a Creative Commons Attribution Licence 4.0.**

For peer review only

*From: Shamseer L, Moher D, Clarke M, Ghersi D, Liberati A, Petticrew M, Shekelle P, Stewart L, PRISMA-P Group. Preferred reporting items for systematic review and meta-analysis protocols (PRISMA-P) 2015: elaboration and explanation. BMJ. 2015 Jan 2;349(jan02 1):g7647.*

For peer review only

Applying dyadic digital psychological interventions for reducing caregiver burden in the illness context: A systematic review and a meta-analysis protocol

The full search strategy that will be further adapted to the following databases: PubMed, Embase, the Cochrane Library, Cinhal, Scopus, PsycINFO, and MEDLINE.

| **Concept Category (combined with AND)** | **Search terms (combined with OR)** |
|---|---|
| Dyads | dyad, dyads, dyadic, couple, couples, spouse, spouses, "Spouse" [MeSH], "Family" [MeSH], informal caregiver, caregiver, "Caregivers" [MeSH], carer, carers, partner |
| Digital | Digital, Online, Internet, internet-based, "Internet"[MeSH], cyberspace, web, web-based, ehealth, e-health, "Telemedicine"[MeSH], mobile health, mhealth, m-health, social media, "Social Media"[MeSH], blog, blogs, mobile app, mobile application, User-Computer Interface, website, webpage |
| Psychological intervention | psychosocial intervention, behavior therapy, "Behavior Therapy" [MeSH], cognitive therapy, "Cognitive Behavioral Therapy" [MeSH], couples therapy, "Couples Therapy" [MeSH], family therapy, "Family Therapy" [MeSH], psychoeducation, psycho-education, psychoeducational, psycho-educational, "Psychology, Medical" [MeSH], "Psychology, Clinical" [MeSH] |

(Dyad* OR Couple* OR "Informal caregiver*" OR Spouse* OR Partner* OR Carer* OR "Care partner*" OR "Informal Carer*") **AND** (Digital OR Online OR "Interne-based" OR Internet OR "Web-based" OR ehealth OR mhealth OR "e-health" OR "m-health" OR "mobile app" OR website OR webpage OR "User-computer Interface") **AND** (Intervention* OR Program* OR Therap* OR Psych* OR "Self-help" OR Support* OR "family-therapy" OR "Cognitive Behavioral Therapy" OR "Behaviour Therapy" or Psychoeducational OR "Psycho-education" OR "Clinical psychology" OR Rehabilitation* OR "Couples therap*") **AND** (RCT OR "randomized controlled trial*) **AND** ("caregiver burden") **NOT** (Parent* OR Child* OR Nurse* OR "Formal caregiv*" OR "Formal carer*")

For peer review only
